# Maximizing Wine Antioxidants: Yeast’s Contribution to Melatonin Formation

**DOI:** 10.3390/antiox13080916

**Published:** 2024-07-29

**Authors:** Elena Cristina Scutarașu, Răzvan George Niță, Laurian Vlase, Cătălin Ioan Zamfir, Bogdan Ionel Cioroiu, Lucia Cintia Colibaba, Dana Muntean, Camelia Elena Luchian, Ana Maria Vlase, Valeriu Cotea

**Affiliations:** 1 Faculty of Horticulture, “Ion Ionescu de la Brad” Iași University of Life Sciences, 3rd M. Sadoveanu Alley, 700490 Iași, Romania; cristina.scutarasu@iuls.ro (E.C.S.); razvannitag@gmail.com (R.G.N.); cintia.colibaba@iuls.ro (L.C.C.); valeriu.cotea@iuls.ro (V.C.); 2 Faculty of Pharmacy, “Iuliu Hațieganu” University of Medicine and Pharmacy, V. Babeș Street, 400000 Cluj Napoca, Romania; laurian.vlase@umfcluj.ro (L.V.); dana.muntean@umfcluj.ro (D.M.); 3 Research Center of Oenology, Romanian Academy, Iași Branch, 9th M. Sadoveanu Alley, 700505 Iași, Romania; catalin.zamfir@acadiasi.ro (C.I.Z.); bogdan.cioroiu@gmail.com (B.I.C.)

**Keywords:** yeasts, white wine, bioactive compounds, increasing nutritional value

## Abstract

Melatonin is commonly found in various fruits, juices, and some fermented beverages. Its concentration in wine is influenced by soil properties, climatic factors, and yeast activity. Even if it is found in fermented beverages in relatively low proportions, melatonin still holds significant nutritional value, giving anti-aging properties, anti-inflammatory actions, and antidepressant effects. In this context, this article focuses on evaluating the impact of different Saccharomyces and non-Saccharomyces yeast species on the formation of melatonin and its contribution to wines’ total antioxidant capacity. Considering that the antioxidant activity of wine is usually related to the content of phenolic compounds, ten such compounds were analyzed. The evaluation of bioactive compounds was performed using high-performance liquid chromatography (HPLC) coupled with mass spectrometry. The total antioxidant capacity of wine samples was evaluated by the ABTS^+^ method. The administration of bâtonnage products increased the efficiency of non-*Saccharomyces* yeasts. The mixtures of *Saccharomyces* and non-*Saccharomyces* yeasts generated higher values for melatonin. The results confirm a significant impact from the grape variety and the specific yeast strains on the melatonin concentration. Also, a strong dependence between antioxidant activity and melatonin levels was observed. Given the limited existing studies on the presence of melatonin in wines, new perspectives are needed for future exploration and understanding.

## 1. Introduction

Melatonin, which is typically synthesized from tryptophan, has received great attention in recent years due to its potential antioxidant activity. In plants, melatonin promotes growth and has shown anti-senescence effects, while in the human body, it helps to improve circadian rhythms, sustain eye health and neurological activity, and has manifested anti-inflammatory, anti-cancer, anti-diabetes, and anti-aging properties. Moreover, melatonin contributes to a general state of well-being, reducing anxiety and depression [1]. The ability to synthesize melatonin in the human body diminishes with age and is influenced by the adopted lifestyle. Thus, diet is an essential factor in the synthesis of different bioactive compounds, including melatonin [2]. This compound can be found in a variety of seeds, cereals, and fruits, including strawberries, blueberries, cherries, sour cherries, and table grapes. It was also identified in drinks, including fruit juices, coffee, tea, beer, and wine [3,4,5,6]. However, relatively few studies have focused on the melatonin content of wine. Although the nutraceutical effects of wine consumption are a controversial subject due to the alcohol content, wine is made up of numerous classes of compounds, many of which have positive effects on the human body. Therefore, the tendency is to produce wines with an improved nutritional value, to counterbalance the negative impact of alcohol. In this context, Boban et al. [7] demonstrated the protective action of wine on the cardiovascular system. In this sense, following an induced myocardial infarction, the survival rate was significantly higher in rats that received reduced amounts of white wine (72.2%), compared to those that received water (47.8%). This effect can be attributed to the numerous biologically active compounds, especially phenolic compounds and melatonin [8,9].

The majority of the existing studies correlate the antiradical activity in wines with their phenolic compound profile [10]. Some research articles that focused on the impact of moderate consumption (1–2 glasses/day) of wine reported a significant increase in the antioxidant capacity in plasma, high-density lipoprotein levels, in parallel with an important decrease in oxidative stress, cardiovascular diseases and cancer cells [11,12]. Different *Saccharomyces* spp. yeasts (*Saccharomyces cerevisiae*, *Saccharomyces uvarum*) and non-*Saccharomyces* spp. (*Candida colliculosa*, *Candida stellata*, *Metschnikowia pulcherima*, *Torulospora delbrueckii*, *Kloeckera thermotolerans*, *Kloeckera apiculata*, *Hanseniaspora uvarum*) were isolated in wine fermentation [13]. In general, non-*Saccharomyces* types of yeast cannot complete alcoholic fermentation and so are frequently used in combination with *Saccharomyces* yeasts. Non-*Saccharomyces* species are able to reduce the initial ethanol level by 1–2% *v*/*v*. Gomez et al. [14] studied the evolution of melatonin and its isomer in the Malbec grape variety using an UHPLC-MS/MS system. Melatonin was identified in the grape extract while its isomer was present in musts and wines. The results confirmed that *Saccharomyces cerevisiae* plays an essential role in the production of melatonin and its isomer in wine. Fernández-Cruz et al. [15] studied melatonin and derived tryptophan metabolites produced during alcoholic fermentation by different *Saccharomyces* and non-*Saccharomyces* (*Torulaspora delbrueckii* and *Metschnikowia pulcherrima*) yeast strains. In Romania, Albu et al. [16] reported for the first time the analysis of melatonin and its precursors. Their study focuses on the development and validation of a sensitive and selective HPLC-MS/MS method for the simultaneous analysis of melatonin, serotonin, and tryptophan in wine samples. Rodriguez-Naranjo et al. [17] did not identify melatonin in Cabernet Sauvignon, Merlot, Syrah, Tempranillo, Tintilla de Rota, Petit Verdot, Pedro Ximénez, Nebbiolo, Palomino Fino. Also, the same results were presented for Flame Seedless, Red Globe, Moscatel Italica, and Superior Seedless, all table grapes from Spain. In a previous study published by the authors [18], melatonin was found in important amounts in table grapes from Romania (Timpuriu de Pietroasa, Coarnă neagră selecționată, Paula). In general, dosages of 0.5 to 5 mg are well tolerated and have no side effects. Although melatonin is naturally present in plants, the quantities are extremely low, making it impossible to obtain concentrations that exceed the maximum permissible concentration, which is the limit for harmful effects on the human body. [19]. Indeed, the concentration of melatonin in fermented beverages is usually low (pg/mL to ng/L), but manifests an important contribution to their nutritional value, which has been less studied in white wines [20]. The increase in the consumption of white wine at the global level requires the development of additional research on the biological effects of white wine [7]. There is increased interest in the finding of natural sources of melatonin and studies are not sufficient in this area. The goal of the present research is to obtain wines that support balanced diets, with high antioxidant capacity. For this reason, this study focuses on optimizing the production technology of some wines by monitoring the influence of different yeasts (*Saccharomyces* and non-*Saccharomyces* yeasts) on the production of melatonin. Since most authors report the antioxidant activity of wines according to the presence of phenolic compounds, ten such compounds were analyzed. To amplify the yeasts’ activity, some bâtonnage products were also applied. The topic is up-to-date and presents novelty through the chosen varieties, but also through the diversity of the inoculated yeasts and applied technology.

## 2. Materials and Methods

### 2.1. Wine Sample

For this experiment, two types of white wines were analyzed: the first category was obtained from an Aligoté + Fetească albă grape blend and the second category from a Sauvignon blanc variety. The grapes were processed using white wine technology, with some particularities. After pressing, the grape juice was transferred into a stainless steel container (600 L) and a dose of 5 g/hL enzyme preparation (Lafase^®^ fruit, Laffort^®^, Bordeaux, France), 80 g/L fining product (Polymust^®^ press, Laffort^®^, Bordeaux, France) and 1 mL/L SO_2_ solution (6%). After 1 day, the lees sediment was evacuated (515 L remained). Different yeast preparations were inoculated in a dose of 20 g/hL, following the producers recommendations: *Lachancea thermotolerans* yeast (CONCERTO^TM^, CHR Hansen, Hørsholm, Denmark); *Saccharomyces cerevisiae* yeast (ZYMAFLORE^®^ X16, Laffort^®^, Bordeaux, France); *Torulaspora delbrueckii* yeast (PRELUDE^TM^, CHR Hansen, Hørsholm, Denmark); *Pichia kluyveri* yeast (FROOTZEN^®^, CHR Hansen, Hørsholm, Denmark); *Saccharomyces cerevisiae* + *Kluyveromyces thermotolerans* yeasts (SYMPHONY^®^, CHR Hansen, Hørsholm, Denmark); *Kluyveromyces thermotolerans* + *Torulaspora delbrueckii* + *Saccharomyces cerevisiae* yeasts (MELODY^TM^, CHR Hansen, Hørsholm, Denmark). After 14 days of fermentation, all the samples were divided into two aliquots. One was directly bottled, while for the second, different bâtonnage products (OENOZYM^®^ TH, Lamothe-Abiet, Canéjan, France—0.06 mL/L; AROMA PROTECT^®^, Lamothe-Abiet, Canéjan, France—0.3 g/L; AROMA T’N’T^®^, Lamothe-Abiet, France—0.3 g/L) were administered and then bottling ensued. The samples were obtained in triplicate and recorded as shown in Table 1.

**Figure 1:**
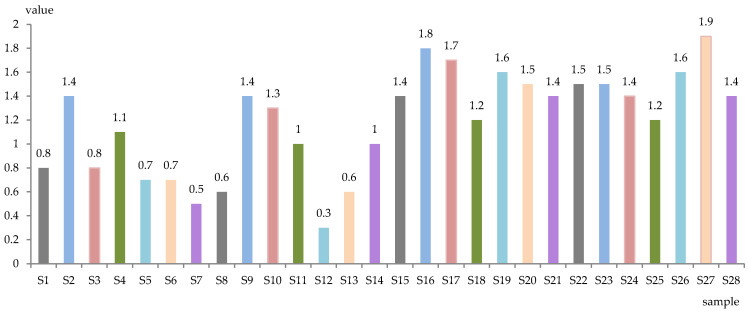
Total sugar of experimental wines (g/L).

**Figure 2:**
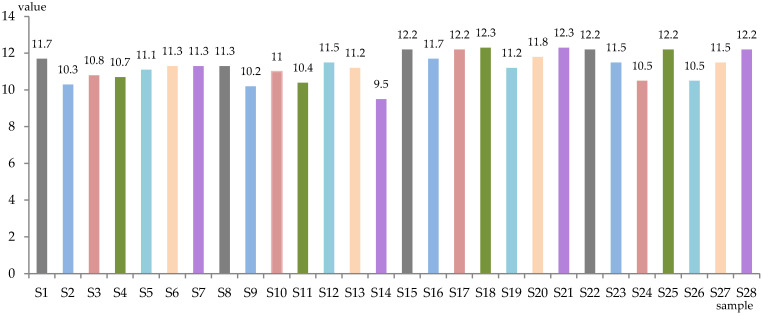
Alcoholic strength of experimental samples (% vol. alc.).

The level of total sugars (g/L) and alcoholic strength (% vol. alc.) of the obtained samples is represented in Figure 1 and Figure 2. The wines were dry, with total sugar content between 0.3 g/L (S12) and 1.9 g/L (S27), while the alcoholic concentration was between 9.5% (S14) and 12.3% (S18, S21) alc. vol.

### 2.2. Laboratory Analysis

For laboratory analysis, standard solutions and different reagents (e.g., melatonin, trans-resveratrol, cis-resveratrol, epicatechin, catechin, gallic acid, protocatechuic acid, caftaric acid, caffeic acid, *p*-coumaric acid, ferulic acid, 6-hydroxy-2,5,7,8-tetramethylchroman-2-carboxylic acid-Trolox solution, 2,2-azinobis(3-ethylbenzothiazoline-6-sulfonate–ABTS^+^ solution, acetonitrile, methanol, isopropanol, acetone, formic acid, triethylamine, hydrogen peroxide, etc.) were purchased from Merck (Darmstadt, Germany).

#### 2.2.1. Phenolic Compound Evaluation

The principal phenolic compounds were determined using an Agilent 1100 HPLC Series system (Agilent, Santa Clara, CA, USA) coupled with an Agilent Ion Trap VL mass spectrometer (Agilent, Santa Clara, USA), following the method presented in our team’s previous papers [21,22]. Wine samples were filtered using 0.45 μm sterile filters. Determinations were performed in triplicate and the results are presented as means, including standard deviations. For the analyzed phenolic compounds, the detection limit (LOD) was 0.04 µg/mL, while the quantification limit (LOQ) was 0.2 µg/mL. Some analytical conditions are presented in Table 2.

#### 2.2.2. Identification and Quantification of Melatonin

This analysis was performed according to the method presented in the previous paper [18], with some modifications and using a Transcend XT Ultimate 3000 UHPLC system (Thermo Scientific TM, Waltham, MA, USA) coupled with a TSQ Access Max mass spectrometer. The elution of compounds was carried out using an Agilent Poroshell C18 column (Agilent, USA) (4.6 × 100 mm, 1.8 μm). The transfer was achieved using a 30% water (TA) and 70% methanol (TD) mixture (*v*/*v*) (in loop). The TX column was prepared using a mixture (*v*/*v*/*v*) of acetonitrile (45%), isopropanol (45%), and acetone (10%) (TB), followed by triethylamine (0.05%) solution prepared in acetonitrile (TC). For separating melatonin from the internal standard, a mobile phase of 0.1% formic acid in water (LA) and 0.1% formic acid in methanol (LB) was used. Regarding the mass spectrometry analysis, a heated electrospray ionization source (in positive mode) was used for ionization, while a collision cell within Q2 was engaged in fragmenting and separating particular ions to allow accurate identification. The ionization conditions were set with an ionization potential of 3 kV, an ionization source temperature of 350 °C, a nebulization gas pressure of 35 psi, and an auxiliary gas pressure of 10 psi. The capillary tube was kept at 350 °C, while the polarity was positive [18]. 

For the standard solutions, a quantity of 10 mg of melatonin was dissolved in methanol and diluted to 10 mL in the same solvent. A volume of 0.5 mL solution was diluted to 10 mL with methanol. A volume of 0.1 mL from the previous solution was diluted with 10 mL of methanol. An internal standard was prepared in water by dissolving a quantity of 6 mg of tryptophan in 10 mL of solvent. A volume of 1.66 mL was diluted to 5 mL with methanol. A series of concentrations of 0.5, 2.0, 10.0, 25.0, and 100 ppb were produced by taking corresponding volumes of the stock solutions, a 0.05 mL internal standard, and dilution to 1 mL with a mixture of water: methanol (50%:50% (*v*/*v*)). For sample preparation, a volume of 2.5 mL of wine was filtered through a 0.45 µm nylon filter; a portion of 1 mL of clear filtrate was spiked with 0.05 mL of internal standard and subjected to analysis according to the method. For the control samples, the second volume of 1.0 mL of filtrate was subjected to analysis to subtract the tryptophan content from the sample. The areas of tryptophan were subtracted from the control samples and used further for the calculation of melatonin in the wine samples. The method’s selectivity was confirmed using blank solutions, showing no interference. Melatonin detection was based on the specific transition of *m*/*z* 233⟶174, with a retention time at 3.28 min. For the internal standard, the detection used the transition of *m*/*z* 205.1⟶146, with a retention time at 2.50 min. A supplementary chromatographic peak was observed in the melatonin chromatogram, likely due to a tryptophan impurity, but it did not interfere with melatonin determination. The method demonstrated linearity in the range of 0.05 ppb to 100 ppb, with calibration points at 0.5, 2.0, 10.0, 25.0, and 100 ppb. The regression correlation coefficient was 0.9945. The back-calculation of standard concentrations using the regression equation showed values within 85% to 115% of the expected concentrations. The highest deviation was 5.85% at 0.5 ppb, and the lowest was 0.7% at 100 ppb. The standard relative deviation for three series of samples under the same conditions was 1.5%. The LOD and LOQ were calculated using the standard deviation of the intercept and the slope, multiplied by 3.3 for LOD and 10 for LOQ. The LOQ was 0.12 ppb, and the LOD was 0.059 ppb, confirmed by the signal-to-noise ratio. Accuracy and precision were evaluated using standard method addition at concentrations of 1 ppb, 25 ppb, and 100 ppb in representative wine samples. Recovery rates were within 85% to 115%: 87.3% at 1 ppb, 92% at 25 ppb, and 93.2% at 100 ppb. Repeatability was assessed with three concentrations within the linearity range, achieving values within 98% to 102% of the target. Inter-day and intra-day precision showed values of 8.5% for the lowest concentration and 6.5% for the highest concentration. The samples were analyzed in triplicate and the results are presented as arithmetic means and standard deviations. The concentration of melatonin is expressed in µg/L [18].

#### 2.2.3. Total Antioxidant Capacity

Total antioxidant capacity of wine samples was evaluated by ABTS^+^ method (also known as Trolox equivalent antioxidant capacity (TEAC) assay), which relies on the ability of antioxidants to diminish the blue-green color of ABTS^+^ in correspondence with their concentrations and scavenging properties. Initially, the reduced ABTS molecule is converted by oxidation to ABTS^+^ using hydrogen peroxide–H_2_O_2_ in an acidic medium of 30 mmol/L acetate buffer solution (pH = 3.6). In the acetate buffer solution, the concentrate (deep green) ABTS^+^ molecules persist for a long time. Another solution of 0.4 mmol/L acetate buffer (pH = 5.8) was prepared and used for the dilution of the initial medium. The color of ABTS^+^ molecules was spontaneously and gradually decolorized. The decolorizing rate is proportional to the concentrations in different antioxidant compounds. The absorbance was monitored at 660 nm and the antioxidant capacity is inversely related to the decolorizing rate of the mixture. The calibration curve was made with Trolox solution and the results are expressed as mmol Trolox equivalent per liter [23].

### 2.3. Statistical Tests

The statistical analysis of the data was carried out using XLStat (Luminevo, Denver, CO, USA) and aimed at the analysis of variance (ANOVA) which reveals the existence of a statistically significant difference (*p*-value < 0.05) between the analyzed samples. Student-*t* test highlights pairs of samples that are significantly different from each other (*p*-value < 0.05). The possible existing correlations between the analyzed bioactive compounds were highlighted by principal components analysis (PCA). Linear regression analysis highlighted the influence of the analyzed bioactive compounds on the antioxidant capacity value.

## 3. Results and Discussion

### 3.1. Effect of Different Yeasts on Wine Bioactive Compounds

According to Table 3 and Table 4, the samples showed different values of bioactive compounds in relation to the specificity of the grape varieties and the applied technology (different species of inoculated yeasts and various bâtonnage products). Tables S1–S3 contains the differences between each pair of samples, for each bioactive compound.

In general, caftaric acid is the main representative in samples obtained from the mix of Aligote + Fetească albă grapes, without bâtonnage (from 16.14 ± 0.15 µg/L in samples with *Lachancea thermotolerans* yeast—S2 to 19.90 ± 0.15 µg/L in S3—*Saccharomyces cerevisiae*). This compound is caffeic acid’s ethyl ester. The results are in accordance to Peréz-Navarro [24] that presented caftaric acid as one of the predominant phenolic acids in white wines. Its concentrations decreased by up to eight times in the case of samples with bâtonnage (from 2.49 ± 0.01 µg/L in S10—*Saccharomyces cerevisiae* yeast + bâtonnage products to 3.28 ± 0.00 µg/L in S11—*Torulaspora delbrueckii* yeast + bâtonnage products). Indeed, bâtonnage (inactivated *Saccharomyces cerevisiae* yeasts, glutathione and pectolytic enzymes) can increase the wines’ complexity and mouthfeel by favoring yeast autolysis and releasing aroma constituents. Other factors that can cause the reduction in phenolic compounds are different chemical and physical processes that can occur, including oxidation, binding to lees, polymerization and precipitation. Certain phenolic molecules have the potential to react with sulfur dioxide, creating more stable complexes [25]. Following bâtonnage application to this category of samples, caffeic acid became predominant in most samples (from 9.31 ± 0.12 µg/L in S11—*Torulaspora delbrueckii* yeast to 10.32 ± 0.01 µg/L in S13—*Saccharomyces cerevisiae* + *Kluyveromyces thermotolerans* yeasts + bâtonnage products). This compound usually derives from *p*-coumaric acid (which results from cinnamic acid), but free forms of caffeic acid can arise due to esterase activity, too [24]. Contrary to these results, this compound was not identified in the white wine samples studied by Onache et al. [26], while a strong positive correlation of caffeic acid with catechin, epicatechin and *trans*-resveratrol was shown by the authors. In the present article, only the positive correlation between epicatechin and catechin was confirmed by principal component analysis, for both Aligoté + Fetească regală (r = 0.937) and Sauvignon blanc wines (r = 0.762).

The concentration of caftaric acid was significantly modified with the inoculation of the analyzed yeast preparations. Significant differences are shown between both yeast and control samples, but each yeast preparation led to significantly different results. For caffeic acid, significantly different concentrations between the following pairs were obtained: S2–S6 (*Lachancea thermotolerans* vs. *Saccharomyces cerevisiae + Kluyveromyces thermotolerans* yeasts), S4–S5 (*Torulaspora delbrueckii* vs. *Pichia kluyveri* yeasts), S5–S6 (*Pichia kluyveri* vs. *Saccharomyces cerevisiae + Kluyveromyces thermotolerans* yeasts) and S5–S7 (*Pichia kluyveri* vs. *Kluyveromyces thermotolerans + Torulaspora delbrueckii + Saccharomyces cerevisiae* yeasts). The differences increased with the application of bâtonnage products. 

Gallic acid showed the highest values in samples obtained from the Sauvignon blanc variety. Garrido and Borges [27] suggested that gallic acid was a significant phenolic compound due to its important scavenging activity. While it can originate from the grape, its presence may also stem from chemical transformations occurring during fermentation. Thus, enzymes and acids present in the grape and microbial activity may catalyze the hydrolysis of hydrolysable and condensed tannins, leading to the release of gallic acid [28]. In this category, samples subjected to bâtonnage (from 20.65 ± 0.03 in S27—*Saccharomyces cerevisiae + Kluyveromyces thermotolerans* yeasts + bâtonnage products to 22.96 ± 0.08 in S23—*Lachancea thermotolerans* yeast + bâtonnage products) with various oenological products indicating slightly higher concentrations compared to those without bâtonnage (from 18.85 ± 0.02 in S16—*Lachancea thermotolerans* yeast, to 20.70 ± 0.20 in S21—*Kluyveromyces thermotolerans + Torulaspora delbrueckii + Saccharomyces cerevisiae* yeasts). If gallic, caffeic, and caftaric acids are predominant in samples without bâtonnage treatment, samples with bâtonnage showed the highest values of gallic acid, caffeic acid, and *p*-coumaric acid. In a separate study conducted by our team [21], it was observed that enzyme preparations had a notable impact on the generation of various phenolic compounds in Sauvignon blanc wines. Among these compounds, protocatechuic acid and caftaric acid were found to be most predominant.

The values recorded in the case of gallic acid are significantly influenced by the applied treatments. Gallic acid is in general influenced by the inoculated yeasts, but minor differences were recorded between S15 and S20 (control sample vs. *Saccharomyces cerevisiae + Kluyveromyces thermotolerans* yeasts), S15 and S21 (control sample vs. *Kluyveromyces thermotolerans + Torulaspora delbrueckii + Saccharomyces cerevisiae* yeasts), S17 and S21 (*Saccharomyces cerevisiae* vs. *Kluyveromyces thermotolerans + Torulaspora delbrueckii + Saccharomyces cerevisiae* yeasts), S20 and S21 (*Saccharomyces cerevisiae + Kluyveromyces thermotolerans* vs. *Kluyveromyces thermotolerans + Torulaspora delbrueckii + Saccharomyces cerevisiae* yeasts). With the exception of samples S5 (*Pichia kluyveri*) and S6 (*Saccharomyces cerevisiae + Kluyveromyces thermotolerans*), all yeast preparations generated significant differences compared to the control sample (S20), in the category of variants without bâtonnage. 

The bâtonnage products in some cases modified gallic acid content, generating significant differences between the control sample and S9 (*Lachancea thermotolerans* yeast + bâtonnage products), as well as S12 (*Pichia kluyveri* yeast + bâtonnage products), S13 (*Saccharomyces cerevisiae + Kluyveromyces thermotolerans* yeasts + bâtonnage products) variants.

The analyzed phenolic acids emerged as significant indicators for distinguishing the analyzed varieties across the diverse wine-growing regions in Romania [29]. Lengyel [30] also noted comparable concentrations of phenolic compounds in wines derived from Sauvignon blanc varieties.

The content of the samples in bioactive compounds was also evaluated after the application of the bâtonnage products, and Table S3 highlights the differences between the pairs of samples with and without this treatment. Therefore, for Aligoté + Fetească albă wines, the most differences were between the variants S1–S8 (control samples) and S2–S9 (*Lachancea thermotolerans* vs. *Lachancea thermotolerans* yeasts + bâtonnage products). Sauvignon blanc samples displayed most differences between the S18–S25 (*Torulaspora delbrueckii*) and S19–S26 (*Pichia kluyveri*) pairs.

Certain strains of yeast have the ability to synthesize melatonin from tryptophan during fermentation, although other microorganisms such as bacteria and fungi may also contribute to its synthesis through enzymatic processes [31,32]. From Table 3 and Table S1, it can certainly be confirmed that the applied technology (different yeasts, application of bâtonnage) influences the melatonin concentration in Aligote + Fetească albă wines. Yeasts administered in samples S2 (*Lachancea thermotolerans* yeast) and S7 (*Kluyveromyces thermotolerans + Torulaspora delbrueckii + Saccharomyces cerevisiae*) did not show significant differences compared to the control sample. With the application of the bâtonnage products, the S14 variant (*Kluyveromyces thermotolerans + Torulaspora delbrueckii + Saccharomyces cerevisiae* yeasts + bâtonnage products) presented a significant difference from S8 (control sample, with bâtonnage, no exogenous yeasts).

Table 4 shows that S15 variant (Sauvignon blanc grape–control sample, no bâtonnage, no exogenous yeasts) presented important levels of melatonin (7.81 ± 0.15 µg/L), followed by S20 (2.71 ± 0.15 µg/L, Sauvignon blanc grapes–*Saccharomyces cerevisiae + Kluyveromyces thermotolerans* yeasts) and S13 (1.58 ± 0.15 µg/L, Aligoté + Fetească albă grapes–*Saccharomyces cerevisiae + Kluyveromyces thermotolerans* yeasts + bâtonnage products), while no melatonin was identified in the S25 sample (Sauvignon blanc grapes-–*Torulaspora delbrueckii* yeasts + bâtonnage products). According to the obtained results, the yeast mix formed by *Saccharomyces cerevisiae + Kluyveromyces thermotolerans* yeasts yielded favorable results in increasing melatonin content for the studied grape varieties. However, it seems that in Sauvignon blanc wines, the control sample showed the maximum identified value (S15—Sauvignon blanc grape control sample, no bâtonnage, no exogenous yeasts). Significant differences were obtained for most pairs of experimental variants, except for S16–S18 (*Lachancea thermotolerans* vs. *Torulaspora delbrueckii* yeasts), S16–C21 (*Lachancea thermotolerans* vs. *Kluyveromyces thermotolerans + Torulaspora delbrueckii + Saccharomyces cerevisiae* yeasts), S17–S19 (*Saccharomyces cerevisiae* vs. *Pichia kluyveri* yeasts) and S18–S21 (*Torulaspora delbrueckii* vs. *Kluyveromyces thermotolerans + Torulaspora delbrueckii + Saccharomyces cerevisiae* yeasts).

According to Fernández-Cruz et al. [15], each yeast type has the ability to produce melatonin at different growth stages. Alcohol content can influence the dilution and release of phenolic compounds and melatonin [31]. For the analyzed samples, minor differences in alcoholic strength were registered. The correlation between melatonin production and growth phase suggests that melatonin may play a role in the yeast’s adaptability to the changing conditions of alcoholic fermentation. Also, melatonin–protein binding for some yeast species should be taken into consideration (not analyzed in this paper) [32]. Fracassetti et al. [33] identified between 0.038 µg/L and 0.063 µg/L melatonin in red wines, being in accordance with the results presented by Vitalini et al. [34]. This compound was found in great amounts (0.011–0.019 µg/mL) in Riesling wines from Romania (commercial samples), analyzed by Albu et al. [16]. In another study, Eremia et al. [8] reported 0.74–0.84 ng/mL melatonin in Fetească neagră and Cabernet sauvignon red wine samples, comparable with the team’s results for white samples. It is clear that different yeasts can synthetize different amounts of bioactive compounds [17]. In accordance with Sunyer-Figueres et al. [35], melatonin acts as a modulator of the biosynthesis of different phenolic compounds. In correlation with Morcillo-Parra et al. [32], melatonin increases the survival of non-*Saccharomyces* species when fermentation is carried out using a mixed inoculum, which is either solely or co-inoculated with non-*Saccharomyces* and *Saccharomyces*. Valera et al. [36] suggested that yeast cells become more fermentative in the presence of melatonin, completing the fermentation a day or two sooner. The authors observed that when melatonin was added to the synthetic must, *Torulaspora delbrueckii* and *Saccharomyces bacillaris* remained until the completion of the fermentation, but *Metschnikowia pulcherrima* and *Hanseniaspora uvarum* only showed up at the start of the process. Therefore, variations in melatonin–protein interactions between non-*Saccharomyces* species may be explained by variations in sugar metabolism and enzyme activity. In another study, Rodriguez-Naranjo et al. [17] evaluated the ability of several *Saccharomyces* and non-*Saccharomyces* yeasts to produce melatonin. Different strains exhibited varying degrees of production; the non-Saccharomyces yeast with the greatest concentration was *Starmerella bacillaris*. However, depending on the yeast strain, extracellular melatonin was found at various stages of the fermentation process. Nevertheless, the same authors also postulated that melatonin requires tryptophan to be present.

**Figure 3:**
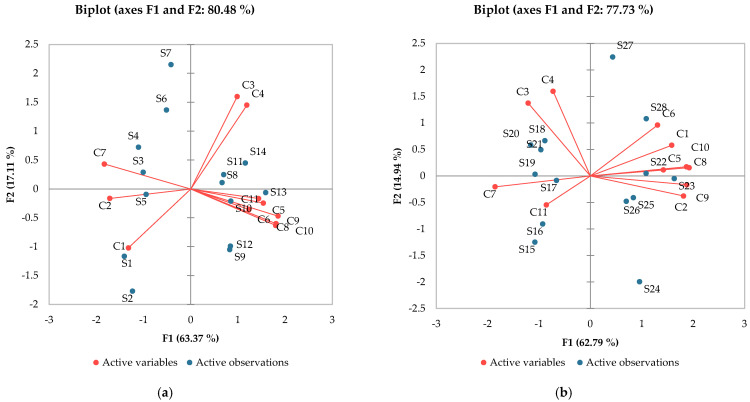
Principal component analysis: (**a**) Aligoté + Fetească albă; (**b**) Sauvignon blanc. C1—*trans*-resveratrol; C2—*cis*-resveratrol; C3—epicatechin; C4—catechin; C5—gallic acid; C6—protocatechuic acid; C7—caftaric acid; C8—caffeic acid; C9—p-coumaric acid; C10—ferulic acid; C11—melatonin. S1–S14—Aligoté + Fetească albă: S1—control sample, no bâtonnage, no exogenous yeasts; S2—*Lachancea thermotolerans* yeast; S3—*Saccharomyces cerevisiae* yeast; S4—*Torulaspora delbrueckii* yeast; S5—*Pichia kluyveri* yeast; S6—*Saccharomyces cerevisiae* + *Kluyveromyces thermotolerans* yeasts; S7—*Kluyveromyces thermotolerans* + *Torulaspora delbrueckii* + *Saccharomyces cerevisiae* yeasts; S8—control sample, with bâtonnage, no exogenous yeasts; S9—*Lachancea thermotolerans* yeast + bâtonnage products; S10—*Saccharomyces cerevisiae* yeast + bâtonnage products; S11—*Torulaspora delbrueckii* yeast + bâtonnage products; S12—*Pichia kluyveri* yeast + bâtonnage products; S13—*Saccharomyces cerevisiae* + *Kluyveromyces thermotolerans* yeasts + bâtonnage products; S14—*Kluyveromyces thermotolerans* + *Torulaspora delbrueckii* + *Saccharomyces cerevisiae* yeasts + bâtonnage products. S15-S28—Sauvignon blanc: S15—control sample, no bâtonnage, no exogenous yeasts; S16—*Lachancea thermotolerans* yeast; S17—*Saccharomyces cerevisiae* yeast; S18—*Torulaspora delbrueckii* yeast; S19—*Pichia kluyveri* yeast; S20—*Saccharomyces cerevisiae* + *Kluyveromyces thermotolerans* yeasts; S21—*Kluyveromyces thermotolerans* + *Torulaspora delbrueckii* + *Saccharomyces cerevisiae* yeasts; S22—control sample, with bâtonnage, no exogenous yeasts; S23—*Lachancea thermotolerans* yeast + bâtonnage products; S24—*Saccharomyces cerevisiae* yeast + bâtonnage products; S25—*Torulaspora delbrueckii* yeast + bâtonnage products; S26—*Pichia kluyveri* yeast + bâtonnage products; S27—*Saccharomyces cerevisiae* + *Kluyveromyces thermotolerans* yeasts + bâtonnage products; S28—*Kluyveromyces thermotolerans* + *Torulaspora delbrueckii* + *Saccharomyces cerevisiae* yeasts + bâtonnage products. The results are significantly influenced by the applied technology when *p*-value is less than 0.05.

For another perspective, principal component analysis (Figure 3) helps to identify the directions of the variation in the results and marks possible correlations between samples and the analyzed compounds. So, as far as melatonin is concerned, the influence of varietal variability was clear. Very high correlations (r > 0.9) between ferulic, *p*-coumaric and caffeic acid could be observed. High correlations (r > 7) were presented by gallic, caffeic, and ferulic acids, while a medium correlation of melatonin and protocatechuic acid was registered (r = 0.610).

The effects of yeasts on the chemical composition of wines have been intensively studied; numerous studies followed the influence of similar oenological products [37,38], but few studies focused on the variation in melatonin content. In general, samples with a high content of melatonin also show higher antioxidant activity, which confirms the results obtained in other studies [18].

### 3.2. Total Antioxidant Activity

**Figure 4:**
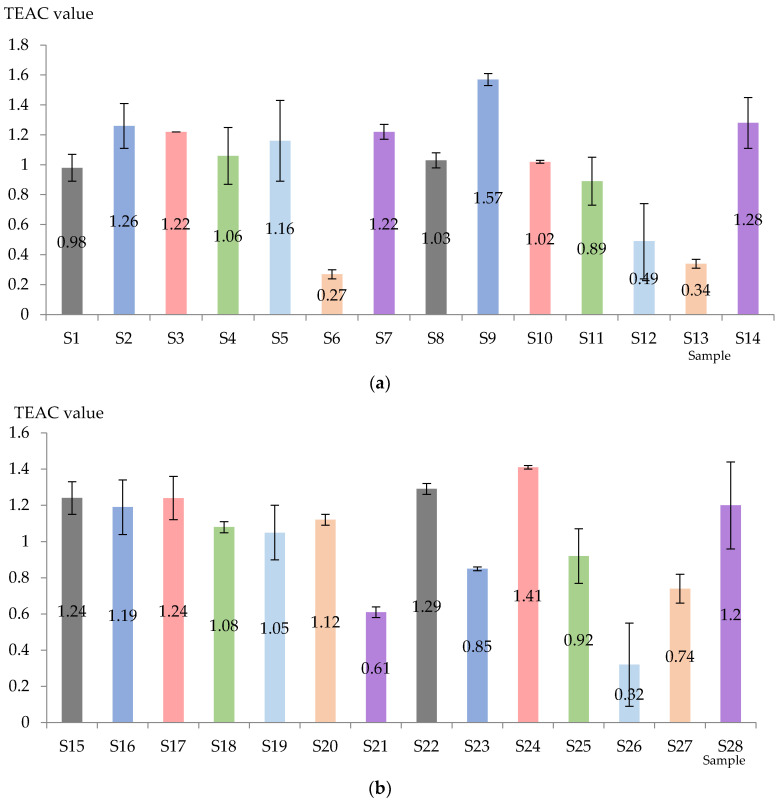
Total antioxidant activity of Aligote + Fetească albă (**a**) and Sauvignon blanc (**b**) wines (mmol Trolox equivalent/L). S1–S14—Aligoté + Fetească albă: S1—control sample, no bâtonnage, no exogenous yeasts; S2—*Lachancea thermotolerans* yeast; S3—*Saccharomyces cerevisiae* yeast; S4—*Torulaspora delbrueckii* yeast; S5—*Pichia kluyveri* yeast; S6—*Saccharomyces cerevisiae* + *Kluyveromyces thermotolerans* yeasts; S7—*Kluyveromyces thermotolerans* + *Torulaspora delbrueckii* + *Saccharomyces cerevisiae* yeasts; S8—control sample, with bâtonnage, no exogenous yeasts; S9—*Lachancea thermotolerans* yeast + bâtonnage products; S10—*Saccharomyces cerevisiae* yeast + bâtonnage products; S11—*Torulaspora delbrueckii* yeast + bâtonnage products; S12—*Pichia kluyveri* yeast + bâtonnage products; S13—*Saccharomyces cerevisiae* + *Kluyveromyces thermotolerans* yeasts + bâtonnage products; S14—*Kluyveromyces thermotolerans* + *Torulaspora delbrueckii* + *Saccharomyces cerevisiae* yeasts + bâtonnage products. S15–S28—Sauvignon blanc: S15—control sample, no bâtonnage, no exogenous yeasts; S16—*Lachancea thermotolerans* yeast; S17—*Saccharomyces cerevisiae* yeast; S18—*Torulaspora delbrueckii* yeast; S19—*Pichia kluyveri* yeast; S20—*Saccharomyces cerevisiae* + *Kluyveromyces thermotolerans* yeasts; S21—*Kluyveromyces thermotolerans* + *Torulaspora delbrueckii* + *Saccharomyces cerevisiae* yeasts; S22—control sample, with bâtonnage, no exogenous yeasts; S23—*Lachancea thermotolerans* yeast + bâtonnage products; S24—*Saccharomyces cerevisiae* yeast + bâtonnage products; S25—*Torulaspora delbrueckii* yeast + bâtonnage products; S26—*Pichia kluyveri* yeast + bâtonnage products; S27—*Saccharomyces cerevisiae* + *Kluyveromyces thermotolerans* yeasts + bâtonnage products; S28—*Kluyveromyces thermotolerans* + *Torulaspora delbrueckii* + *Saccharomyces cerevisiae* yeasts + bâtonnage products. The results are significantly influenced by the applied technology when *p*-value is less than 0.05.

The TEAC value was obviously influenced by the applied technology and the variability of the variety (Figure 4). For Aligoté + Fetească albă (without bâtonnage) the highest value was recorded in the case of S2 (*Lachancea thermotolerans* yeast), followed by S7 (*Kluyveromyces thermotolerans + Torulaspora delbrueckii + Saccharomyces cerevisiae* yeasts), S3 (*Saccharomyces cerevisiae* yeast), and the lowest value was obtained with S6 (*Saccharomyces cerevisiae + Kluyveromyces thermotolerans* yeasts). When bâtonnage was applied, the order was as follows: S9 (*Lachancea thermotolerans* yeast) > S14 (*Kluyveromyces thermotolerans + Torulaspora delbrueckii + Saccharomyces cerevisiae* yeasts + bâtonnage products) > S8 (control sample, with bâtonnage, no exogenous yeasts) > S10 (*Saccharomyces cerevisiae* yeast + bâtonnage products) > S11 (*Torulaspora delbrueckii* yeast + bâtonnage products) > S2 (*Lachancea thermotolerans* yeast) > S13 *Saccharomyces cerevisiae + Kluyveromyces thermotolerans* yeasts + bâtonnage products). It can be seen that *Lachancea thermotolerans* yeast was the most effective in increasing the TEAC value, in contrast to the mixture *Saccharomyces cerevisiae + Kluyveromyces thermotolerans* yeasts.

Sauvignon blanc wines are generally characterized by higher TEAC values. In the samples without bâtonnage, the highest antioxidant activity was suggested for S15 (control sample, no bâtonnage, no exogenous yeasts), followed by S17 (*Saccharomyces cerevisiae* yeast) and S8 (control sample, with bâtonnage, no exogenous yeasts), and the lowest value was found in S21 (*Kluyveromyces thermotolerans + Torulaspora delbrueckii + Saccharomyces cerevisiae* yeasts). On the other hand, S24 (*Saccharomyces cerevisiae* yeast) and S22 (control sample, with bâtonnage, no exogenous yeasts) were highlighted as having the highest TEAC values, while S27 (*Saccharomyces cerevisiae + Kluyveromyces thermotolerans* yeasts) and S26 recorded the lowest (*Pichia kluyveri* yeast + bâtonnage products). It was observed that samples without bâtonnage generally had a higher TEAC value. Favorable results were also obtained by the yeasts inoculated in S2 (*Lachancea thermotolerans*) and S3 (*Saccharomyces cerevisiae*). The higher value of TEAC could suggest a higher antioxidant activity, thus indicating better oxidative stability, improved nutritional value and even potential health benefits.

The results obtained from linear regression (Table 5 and Table 6) indicate that over 90% of the variability of the dependent variables (TEAC) is explained by the explanatory variable (bioactive compounds) for each groups of samples (91% for Aligoté + Fetească albă without bâtonnage, 94% for Aligoté + Fetească albă with bâtonnage, 94% for Sauvignon blanc without bâtonnage, 98% for Sauvignon blanc with bâtonnage). This fact suggests that the analyzed compounds are generally the main antioxidants in white wines, at least those analyzed in this study.

In the case of Aligoté + Fetească albă samples, compounds such as *trans*-resveratrol, catechin, gallic acid, *p*-coumaric acid, and caftaric acid showed a negative influence on the TEAC value, suggesting a lower antioxidant activity. On the other hand, melatonin followed by *cis*-resveratrol, ferulic acid, protocatechuic acid, caffeic and epicatechin, presented a positive contribution to the increase in the TEAC value, indicating a greater antioxidant activity. For the second category, wines with bâtonnage, *cis*-resveratrol, protocatechuic acid, caffeic acid showed a negative impact on the TEAC value, while melatonin > catechin > *p*-coumaric > gallic acid > ferulic acid > *trans*-resveratrol > caftaric acid showed a positive contribution on the TEAC value.

In Sauvignon blanc wines, the TEAC value was negatively influenced by the concentration of compounds such as gallic, protocatechuic, caftaric, caffeic, *p*-coumaric, and ferulic acids. Also, the higher positive contribution was evident for melatonin, followed by *cis*-resveratrol, epicatechin, catechin, and *trans*-resveratrol. After the administration of bâtonnage products, melatonin exhibited the greatest influence, followed by *p*-coumaric, protocatechuic, *cis*-resveratrol and ferulic acids.

Therefore, the hypothesis is confirmed that although it is found in very small proportions in wines, melatonin may have made the largest contribution to antioxidant activity in the analyzed samples. Similar results have been reported previously. According to Sunyer-Figueres [35], melatonin exhibits direct antioxidant action (by eliminating reactive oxygen species) and indirect (by decreasing oxidized glutathione and activating genes involved in the response to oxidative stress such as catalase, glutathione, glutaredoxin and thioredoxin). Also, the authors postulated that melatonin confers protection against ethanol stress. Melatonin may act synergistically with other wine antioxidants, resulting in an increased cytoprotective impact against oxidative stress [8,9]. The results are in accordance with Vasquez et al. [39], confirming that melatonin manifests important anti-scavenging action on *Saccharomyces cerevisiae* yeast but, in the present study, the results showed a better efficiency when *Saccharomyces cerevisiae* yeast was inoculated in combination with non-*Saccharomyces* species.

There are numerous chemical, environmental, and methodological elements that can interact with phenolic acids in wine and may influence their contribution to total antioxidant activity. These interactions can also have positive or negative oxidative effects. Therefore, phenolic acids can form complexes with other wine components, such as proteins, metals, or other phenolic compounds. Consequently, phenolic acids have the ability to form complexes with other elements found in wine, including proteins, metals, and other phenolic compounds. These complexes have the potential to modify the phenolic acids’ availability or reactivity, which might impact their antioxidant efficacy. These complexes may occasionally promote oxidation processes as opposed to inhibiting them. Phenolic acids’ antioxidant activity can change depending on the pH and external factors like temperature and oxygen exposure. For instance, phenolic acids’ ionization state and reactivity can change in response to pH changes, which might impact their capacity to scavenge free radicals and contribute hydrogen atoms or electrons. The assessment of total antioxidant activity may also be impacted by the analysis technique adopted [40]. This might be due to side reactions such as the formation of coupling adducts with ABTS^+^ by different phenolic acids or a pro-oxidation reaction. Variations in the reported effects of phenolic acids may result from various assays that capture different features of antioxidant capacity or are more sensitive to particular types of antioxidants [40].

The presented results show that the antioxidant action of melatonin is dependent on various factors, such as the variability of the variety, the chemical composition and the applied technology. These variations may have an impact on the interactions of melatonin in each grape variety. It is important to explore more about the distinct qualities of each wine variety, their individual compositions, and the ways in which melatonin interacts with those components to explain the variations in its contribution to antioxidant activity that have been found. 

## 4. Conclusions

The findings reported in this article indicate that the production of health-promoting compounds depends not only on a strain-specific property of the yeasts in the environment, but also on the varietal characteristics of the grape. Choosing the appropriate strain of primary yeasts is an effective way to enrich wines with health-promoting compounds other than the well-known and much-studied phenolic compounds. Melatonin and phenolic compounds play a significant role in defining the antioxidant activity of wines. The analyzed wine samples displayed different variations in the concentration of bioactive compounds, depending on the type of inoculated yeasts. For Aligoté + Fetească albă samples, caftaric acid was predominant in samples where *Saccharomyces cerevisiae* yeast was inoculated. The administration of bâtonnage products generated better efficiency from the *Torulaspora delbrueckii* yeast for this compound. Melatonin was found in higher amounts in samples where *Pichia kluyveri* yeast was inoculated, while the use of bâtonnage products led to increased levels in the *Saccharomyces cerevisiae* + *Kluyveromyces thermotolerans* mix. The blend of *Kluyveromyces thermotolerans* + *Torulaspora delbrueckii* + *Saccharomyces cerevisiae* yeasts generated the highest concentrations of gallic acid in Sauvignon blanc wines. For this category of samples, melatonin was predominant in the control sample, where no treatment was applied, while bâtonnage addition generated an increased content for this compound in samples where *Kluyveromyces thermotolerans* + *Torulaspora delbrueckii* + *Saccharomyces cerevisiae* yeasts were added. The administered bâtonnage products increased the efficiency of *Lachancea thermotolerans* yeasts in obtaining higher values for *cis*-resveratrol, caftaric, caffeic and ferulic acids. In general, samples with higher melatonin values also showed important concentrations of phenolic compounds. It can thus be concluded that the antioxidant properties of melatonin contribute to the stability of phenolic compounds, helping to maintain their concentration and biological activity in wine. Although found in low concentrations in wine, the increase in antioxidant capacity is significantly dependent on the value of this compound. The contribution of yeasts to the production of melatonin is still poorly investigated, so future research is needed.

## Figures and Tables

**Table 1:** Sample codifications.

Aligoté+ Fetească Albă	Sauvignon Blanc	Bâtonnage Treatment	Inoculated Yeasts	Yeasts’ Alcohol Tolerance *
S1	S15	Control sample, no bâtonnage, no exogenous yeasts		
S2	S16	No bâtonnage	*Lachancea thermotolerans*	10%
S3	S17		*Saccharomyces cerevisiae*	13%
S4	S18		*Torulaspora delbrueckii*	11%
S5	S19		*Pichia kluyveri*	4–5%
S6	S20		*Saccharomyces cerevisiae* + *Kluyveromyces thermotolerans*	16%
S7	S21		*Kluyveromyces thermotolerans* + *Torulaspora delbrueckii* + *Saccharomyces cerevisiae*	15%
S8	S22	Control sample, with bâtonnage, no exogenous yeasts		
S9	S23	With bâtonnage products	*Lachancea thermotolerans*	10%
S10	S24		*Saccharomyces cerevisiae*	13%
S11	S25		*Torulaspora delbrueckii*	11%
S12	S26		*Pichia kluyveri*	4–5%
S13	S27		*Saccharomyces cerevisiae* + *Kluyveromyces thermotolerans*	16%
S14	S28		*Kluyveromyces thermotolerans* + *Torulaspora delbrueckii* + *Saccharomyces cerevisiae*	15%

* Values are presented according to the producers’ technical sheets.

**Table 2:** Analytical parameters applied for the detection and quantification of phenolic compounds [21,22].

Phenolic Compounds	Retention Time (min.)	Specific Ions
*trans*-resveratrol	1.5	229 > 134.9
*cis*-resveratrol	2.3	229 > 134.9
epicatechin	9.0	289
catechin	6.0	289
gallic acid	1.5	169
protocatechuic acid	2.8	153
caftaric acid	3.54	311 > 148.6, 178.6
caffeic acid	5.60	179.4 > 134.7
*p*-coumaric acid	9.48	163 > 118.7
ferulic acid	12.8	193.2 > 133.7, 148.7, 177.6

**Table 3:** Bioactive compounds in wine samples obtained from Aligoté + Fetească albă wines.

Sample	C1	C2	C3	C4	C5	C6	C7	C8	C9	C10	C11
Unit Measure	µg/mL										µg/L
S1	0.38 ± 0.08	0.68 ± 0.02	0.47 ± 0.01	0.34 ± 0.04	1.83 ± 0.04	0.32 ± 0.01	19.54 ± 0.00	1.51 ± 0.45	0.61 ± 0.24	0.18 ± 0.12	0.22 ± 0.10
S2	0.51 ± 0.05	0.61 ± 0.10	0.48 ± 0.05	0.33 ± 0.00	1.45 ± 0.01	0.46 ± 0.03	16.65 ± 0.10	1.75 ± 0.12	0.97 ± 0.08	0.35 ± 0.05	0.32 ± 0.02
S3	0.39 ± 0.02	0.64 ± 0.02	0.70 ± 0.25	0.53 ± 0.05	1.62 ± 0.01	0.36 ± 0.00	19.90 ± 0.05	1.64 ± 0.11	0.64 ± 0.05	0.20 ± 0.02	0.70 ± 0.05
S4	0.30 ± 0.01	0.61 ± 0.05	0.74 ± 0.01	0.49 ± 0.15	1.65 ± 0.08	0.26 ± 0.01	16.14 ± 0.15	1.51 ± 0.10	0.55 ± 0.00	0.18 ± 0.10	0.03 ± 0.00
S5	0.40 ± 0.05	0.59 ± 0.10	0.67 ± 0.02	0.48 ± 0.01	1.74 ± 0.00	0.32 ± 0.01	17.13 ± 0.04	1.79 ± 0.04	0.58 ± 0.15	0.18 ± 0.12	0.81 ± 0.03
S6	0.28 ± 0.01	0.47 ± 0.08	0.81 ± 0.00	0.60 ± 0.00	1.81 ± 0.02	0.33 ± 0.04	17.68 ± 0.00	1.52 ± 0.05	0.61 ± 0.30	0.18 ± 0.04	0.45 ± 0.01
S7	0.24 ± 0.10	0.58 ± 0.00	0.94 ± 0.05	0.66 ± 0.02	1.82 ± 0.00	0.45 ± 0.00	18.59 ± 0.12	1.57 ± 0.10	0.62 ± 0.08	0.17 ± 0.15	0.24 ± 0.02
***p*-values**	0.001	0.042	0.000	<0.0001	<0.0001	<0.0001	<0.0001	0.389	0.094	0.312	<0.0001
S8	0.11 ± 0.04	0.48 ± 0.01	0.73 ± 0.01	0.52 ± 0.00	1.89 ± 0.04	0.42 ± 0.00	2.97 ± 0.03	8.99 ± 0.11	1.51 ± 0.12	0.63 ± 0.00	0.65 ± 0.00
S9	0.32 ± 0.06	0.44 ± 0.02	0.66 ± 0.04	0.51 ± 0.08	2.35 ± 0.00	0.42 ± 0.02	3.09 ± 0.06	10.08 ± 0.08	1.70 ± 0.01	0.81 ± 0.02	0.50 ± 0.01
S10	0.26 ± 0.03	0.50 ± 0.04	0.75 ± 0.00	0.60 ± 0.00	1.93 ± 0.20	0.40 ± 0.04	2.49 ± 0.01	10.27 ± 0.04	1.79 ± 0.10	0.84 ± 0.15	0.87 ± 0.01
S11	0.26 ± 0.20	0.46 ± 0.05	0.85 ± 0.10	0.56 ± 0.05	1.89 ± 0.10	0.45 ± 0.01	3.28 ± 0.00	9.31 ± 0.12	1.67 ± 0.05	0.71 ± 0.08	0.35 ± 0.03
S12	0.34 ± 0.10	0.45 ± 0.00	0.72 ± 0.01	0.50 ± 0.04	2.13 ± 0.02	0.47 ± 0.05	2.77 ± 0.03	10.12 ± 0.04	1.73 ± 0.02	0.68 ± 0.12	1.10 ± 0.15
S13	0.22 ± 0.00	0.47 ± 0.04	0.82 ± 0.01	0.64 ± 0.00	2.20 ± 0.05	0.65 ± 0.00	2.77 ± 0.04	10.32 ± 0.01	1.79 ± 0.12	0.87 ± 0.00	1.58 ± 0.10
S14	0.18 ± 0.12	0.36 ± 0.11	0.79 ± 0.08	0.62 ± 0.05	1.92 ± 0.01	0.56 ± 0.02	2.85 ± 0.04	9.40 ± 0.11	1.64 ± 0.35	0.73 ± 0.01	0.62 ± 0.05
*p*-values	0.153	0.092	0.007	0.005	<0.0001	<0.0001	<0.0001	<0.0001	0.356	0.019	<0.0001

C1—*trans*-resveratrol; C2—*cis*-resveratrol; C3—epicatechin; C4—catechin; C5—gallic acid; C6—protocatechuic acid; C7—caftaric acid; C8—caffeic acid; C9—*p*-coumaric acid; C10—ferulic acid; C11—melatonin; S1—control sample, no bâtonnage, no exogenous yeasts; S2—*Lachancea thermotolerans* yeast; S3—*Saccharomyces cerevisiae* yeast; S4—*Torulaspora delbrueckii* yeast; S5—*Pichia kluyveri* yeast; S6—*Saccharomyces cerevisiae* + *Kluyveromyces thermotolerans* yeasts; S7—*Kluyveromyces thermotolerans* + *Torulaspora delbrueckii* + *Saccharomyces cerevisiae* yeasts; S8—control sample, with bâtonnage, no exogenous yeasts; S9—*Lachancea thermotolerans* yeast + bâtonnage products; S10—*Saccharomyces cerevisiae* yeast + bâtonnage products; S11—*Torulaspora delbrueckii* yeast + bâtonnage products; S12—*Pichia kluyveri* yeast + bâtonnage products; S13—*Saccharomyces cerevisiae* + *Kluyveromyces thermotolerans* yeasts + bâtonnage products; S14—*Kluyveromyces thermotolerans* + *Torulaspora delbrueckii* + *Saccharomyces cerevisiae* yeasts + bâtonnage products. The results are significantly influenced by the applied technology when *p*-value is less than 0.05.

**Table 4:** Bioactive compounds in Sauvignon blanc wine samples.

Sample	C1	C2	C3	C4	C5	C6	C7	C8	C9	C10	C11
Unit Measure	µg/mL										µg/L
S15	0.50 ± 0.07	0.89 ± 0.02	0.20 ± 0.04	0.14 ± 0.04	20.62 ± 0.01	0.37 ± 0.03	3.96 ± 0.00	3.42 ± 0.12	0.31 ± 0.15	0.21 ± 0.05	7.81 ± 0.15
S16	0.48 ± 0.02	0.91 ± 0.08	0.22 ± 0.03	0.12 ± 0.04	18.85 ± 0.02	0.42 ± 0.01	3.72 ± 0.02	3.56 ± 0.15	0.31 ± 0.10	0.25 ± 0.04	0.46 ± 0.05
S17	0.50 ± 0.01	1.12 ± 0.02	0.22 ± 0.04	0.16 ± 0.01	20.67 ± 0.00	0.39 ± 0.00	3.68 ± 0.05	3.69 ± 0.00	0.58 ± 0.08	0.35 ± 0.20	0.69 ± 0.03
S18	0.53 ± 0.20	0.77 ± 0.0	0.26 ± 0.02	0.15 ± 0.00	19.69 ± 0.03	0.53 ± 0.03	3.64 ± 0.04	3.62 ± 0.02	0.40 ± 0.15	0.30 ± 0.05	0.40 ± 0.10
S19	0.37 ± 0.05	0.88 ± 0.00	0.22 ± 0.01	0.17 ± 0.04	19.78 ± 0.02	0.35 ± 0.04	3.44 ± 0.04	3.62 ± 0.08	0.34 ± 0.05	0.20 ± 0.15	0.65 ± 0.08
S20	0.27 ± 0.02	0.66 ± 0.02	0.27 ± 0.02	0.17 ± 0.04	20.64 ± 0.01	0.41 ± 0.03	3.99 ± 0.10	3.51 ± 0.01	0.58 ± 0.02	0.38 ± 0.10	2.71 ± 0.15
S21	0.36 ± 0.04	0.93 ± 0.05	0.26 ± 0.01	0.17 ± 0.03	20.70 ± 0.20	0.35 ± 0.00	4.11 ± 0.20	3.47 ± 0.05	0.55 ± 0.15	0.43 ± 0.20	0.41 ± 0.05
*p*-values	0.016	<0.0001	0.042	0.448	<0.0001	<0.0001	<0.0001	0.014	0.017	0.296	<0.0001
S22	1.01 ± 0.05	1.39 ± 0.03	0.16 ± 0.05	0.14 ± 0.02	21.11 ± 0.03	0.64 ± 0.02	0.24 ± 0.04	5.93 ± 0.20	2.00 ± 0.08	0.76 ± 0.05	0.37 ± 0.15
S23	0.99 ± 0.00	1.79 ± 0.00	0.16 ± 0.04	0.14 ± 0.01	22.96 ± 0.08	0.61 ± 0.01	0.51 ± 0.05	6.67 ± 0.04	2.30 ± 0.10	0.96 ± 0.04	0.21 ± 0.02
S24	0.43 ± 0.01	1.72 ± 0.03	0.16 ± 0.03	0.11 ± 0.00	20.93 ± 0.20	0.34 ± 0.01	0.47 ± 0.18	6.17 ± 0.01	2.72 ± 0.05	0.94 ± 0.00	0.26 ± 0.03
S25	0.80 ± 0.03	1.36 ± 0.04	0.21 ± 0.01	0.12 ± 0.01	21.21 ± 0.03	0.47 ± 0.02	0.28 ± 0.04	5.80 ± 0.10	2.06 ± 0.02	0.91 ± 0.15	0.22 ± 0.00
S26	0.67 ± 0.04	1.29 ± 0.05	0.17 ± 0.00	0.13 ± 0.04	20.70 ± 0.03	0.54 ± 0.03	0.24 ± 0.22	5.56 ± 0.08	1.97 ± 0.15	0.74 ± 0.04	0.25 ± 0.05
S27	0.77 ± 0.00	1.30 ± 0.00	0.28 ± 0.01	0.18 ± 0.0	20.65 ± 0.08	0.60 ± 0.01	0.00 ± 0.00	6.02 ± 0.05	1.45 ± 0.04	0.81 ± 0.00	0.28 ± 0.08
S28	1.21 ± 0.01	1.32 ± 0.00	0.22 ± 0.05	0.16 ± 0.02	21.22 ± 0.05	0.56 ± 0.03	0.28 ± 0.05	6.24 ± 0.02	2.51 ± 0.08	0.94 ± 0.01	0.52 ± 0.04
*p*-values	<0.0001	<0.0001	0.004	0.016	<0.0001	<0.0001	0.015	<0.0001	<0.0001	0.002	<0.0001

C1—*trans*-resveratrol; C2—*cis*-resveratrol; C3—epicatechin; C4—catechin; C5—gallic acid; C6—protocatechuic acid; C7—caftaric acid; C8—caffeic acid; C9—*p*-coumaric acid; C10—ferulic acid; C11—melatonin; S15—control sample, no bâtonnage, no exogenous yeasts; S16—*Lachancea thermotolerans* yeast; S17—*Saccharomyces cerevisiae* yeast; S18—*Torulaspora delbrueckii* yeast; S19—*Pichia kluyveri* yeast; S20—*Saccharomyces cerevisiae* + *Kluyveromyces thermotolerans* yeasts; S21—*Kluyveromyces thermotolerans* + *Torulaspora delbrueckii* + *Saccharomyces cerevisiae* yeasts; S22—control sample, with bâtonnage, no exogenous yeasts; S23—*Lachancea thermotolerans* yeast + bâtonnage products; S24—*Saccharomyces cerevisiae* yeast + bâtonnage products; S25—*Torulaspora delbrueckii* yeast + bâtonnage products; S26—*Pichia kluyveri* yeast + bâtonnage products; S27—*Saccharomyces cerevisiae* + *Kluyveromyces thermotolerans* yeasts + bâtonnage products; S28—*Kluyveromyces thermotolerans* + *Torulaspora delbrueckii* + *Saccharomyces cerevisiae* yeasts + bâtonnage products. The results are significantly influenced by the applied technology when *p*-value is less than 0.05.

**Table 5:** The contribution of each bioactive compound on total antioxidant capacity of Aligoté + Fetească albă wines.

Source	Beta Coefficient	Standard Error	t	Pr > |t|	Lower Bound (95%)	Upper Bound (95%)	Value	Standard Error	t	Pr > |t|	Lower Bound (95%)	Upper Bound (95%)
	Without bâtonnage						With bâtonnage					
C1	−5.593	4.276	−1.308	0.223 °	−15.266	4.081	0.697	0.611	1.142	0.283 °	−0.684	2.078
C2	3.961	0.619	6.396	0.000 ***	2.560	5.362	−5.568	3.293	−1.691	0.125 °	−13.017	1.881
C3	0.110	0.481	0.228	0.824 °	−0.978	1.197	−2.791	1.653	−1.688	0.126 °	−6.531	0.949
C4	−2.121	2.063	−1.028	0.331 °	−6.787	2.546	7.070	1.675	4.222	0.002 *	3.282	10.859
C5	−1.744	0.579	−3.011	0.015 *	−3.054	−0.434	1.573	0.792	1.987	0.078 .	−0.218	3.364
C6	2.584	0.771	3.352	0.008 **	0.840	4.328	−6.185	3.654	−1.692	0.125 °	−14.452	2.082
C7	−0.002	0.049	−0.033	0.975 °	−0.112	0.109	0.178	0.374	0.476	0.645 °	−0.668	1.025
C8	1.449	0.555	2.612	0.028 *	0.194	2.704	−1.100	0.396	−2.781	0.021 *	−1.995	−0.205
C9	−2.062	0.599	−3.443	0.007 **	−3.416	−0.707	1.937	0.863	2.245	0.051 .	−0.015	3.889
C10	3.458	2.819	1.227	0.251 °	−2.919	9.835	1.054	0.790	1.334	0.215 °	−0.733	2.842
C11	362.824	431.340	0.841	0.422 °	−612.934	1338.583	271.168	675.415	0.401	0.697 °	−1256.727	1799.062

C1—*trans*-resveratrol; C2—*cis*-resveratrol; C3—epicatechin; C4—catechin; C5—gallic acid; C6—protocatechuic acid; C7—caftaric acid; C8—caffeic acid; C9—*p*-coumaric acid; C10—ferulic acid; C11—melatonin. The antioxidant capacity is significantly influenced by the analyzed bioactive compound when *p*-value is less than 0.05: 0 < *** < 0.001 < ** < 0.01 < * < 0.05 < . < 0.1 < ° < 1.

**Table 6:** The contribution of each bioactive compound on total antioxidant capacity of Sauvignon blanc wines.

Source	Beta Coefficient	Standard Error	t	Pr > |t|	Lower Bound (95%)	Upper Bound (95%)	Value	Standard Error	t	Pr > |t|	Lower Bound (95%)	Upper Bound (95%)
	Without bâtonnage						With bâtonnage					
C1	−0.856	0.157	−5.442	0.000 ***	−1.212	−0.500	5.372	1.082	4.963	0.001 ***	2.924	7.820
C2	0.652	0.200	3.265	0.010 **	0.200	1.103	10.197	2.431	4.194	0.002 **	4.697	15.697
C3	−0.460	0.137	−3.357	0.008 **	−0.770	−0.150	7.216	2.710	2.663	0.026 *	1.086	13.346
C4	−0.092	0.161	−0.570	0.582 °	−0.457	0.273	5.486	4.511	1.216	0.255 °	−4.719	15.690
C5	−1.027	0.169	−6.079	0.000 ***	−1.410	−0.645	−0.849	0.177	−4.808	0.001 ***	−1.249	−0.450
C6	0.836	0.200	4.170	0.002 **	0.382	1.289	−4.380	1.699	−2.578	0.030 *	−8.224	−0.536
C7	−1.061	0.217	−4.884	0.001 ***	−1.552	−0.569	−0.899	0.962	−0.934	0.374 °	−3.077	1.278
C8	−0.185	0.329	−0.562	0.588 °	−0.929	0.559	−3.338	1.248	−2.674	0.025 *	−6.161	−0.514
C9	1.331	0.294	4.533	0.001 **	0.667	1.995	−0.275	0.455	−0.603	0.561 °	−1.304	0.755
C10	0.398	0.126	3.162	0.012 *	0.113	0.683	−0.523	0.785	−0.666	0.522 °	−2.298	1.252
C11	1.643	0.122	13.462	<0.0001 ***	1.367	1.920	792.310	489.546	1.618	0.140 °	−315.120	1899.741

C1—*trans*-resveratrol; C2—*cis*-resveratrol; C3—epicatechin; C—catechin; C5—gallic acid; C6—protocatechuic acid; C7—caftaric acid; C8—caffeic acid; C9—*p*-coumaric acid; C10—ferulic acid; C11—melatonin. The antioxidant capacity is significantly influenced by the analyzed bioactive compound when *p*-value is less than 0.05: 0 < *** < 0.001 < ** < 0.01 < * < 0.05 < 0.1 < ° < 1.

## Data Availability

The raw data supporting the conclusions of this article will be made available by the authors on request.

## Supplementary Materials

The following supporting information can be downloaded at: https://www.mdpi.com/article/10.3390/antiox13080916/s1, Table S1: Student’s *t*-test for Fetească regală+Aligoté wines; Table S2: Student’s *t*-test for Sauvignon blanc wines; Table S3: *t*-test between samples without and with bâtonnage.
