# Supplementary material for: Maximizing Wine Antioxidants: Yeast’s Contribution to Melatonin Formation
Source: Antioxidants (Basel). 2024 Jul 29;13(8):916. doi: 10.3390/antiox13080916 (PMC11351232; doi:10.3390/antiox13080916)
Supplement: Supplementary file 1 [file antioxidants-13-00916-s001.zip › antioxidants-3089882-supplementary.pdf]

Table S1. Student's t-test for Fetească regală+Aligoté wines.

Without bâtonnage products								With bâtonnage products							
**Sample**	**S1**	**S2**	**S3**	**S4**	**S5**	**S6**	**S7**	**Sample**	**S10**	**S11**	**S12**	**S13**	**S14**	**S8**	**S9**
								**C1**							
**S1**	1	0.074	0.748	0.2	0.699	0.116	0.143	**S10**	1	0.995	0.22	0.082	0.329	0.007	0.185
**S2**	0.074	1	0.021	0.002	0.057	0.002	0.015	**S11**	0.995	1	0.531	0.752	0.592	0.281	0.63
**S3**	0.748	0.021	1	0.002	0.852	0.001	0.062	**S12**	0.22	0.531	1	0.092	0.138	0.019	0.722
**S4**	0.2	0.002	0.002	1	0.033	0.057	0.338	**S13**	0.082	0.752	0.092	1	0.601	0.01	0.042
**S5**	0.699	0.057	0.852	0.033	1	0.017	0.072	**S14**	0.329	0.592	0.138	0.601	1	0.404	0.142
**S6**	0.116	0.002	0.001	0.057	0.017	1	0.514	**S8**	0.007	0.281	0.019	0.01	0.404	1	0.007
**S7**	0.143	0.015	0.062	0.338	0.072	0.514	1	**S9**	0.185	0.63	0.722	0.042	0.142	0.007	1
								**C2**							
**S1**	1	0.321	0.083	0.086	0.226	0.013	0.001	**S10**	1	0.346	0.133	0.455	0.111	0.542	0.085
**S2**	0.321	1	0.647	0.955	0.841	0.139	0.673	**S11**	0.346	1	0.895	0.754	0.232	0.468	0.565
**S3**	0.083	0.647	1	0.348	0.473	0.026	0.009	**S12**	0.133	0.895	1	0.516	0.212	0.009	0.252
**S4**	0.086	0.955	0.348	1	0.843	0.072	0.482	**S13**	0.455	0.754	0.516	1	0.177	0.663	0.284
**S5**	0.226	0.841	0.473	0.843	1	0.184	0.887	**S14**	0.111	0.232	0.212	0.177	1	0.129	0.289
**S6**	0.013	0.139	0.026	0.072	0.184	1	0.076	**S8**	0.542	0.468	0.009	0.663	0.129	1	0.029
**S7**	0.001	0.673	0.009	0.482	0.887	0.076	1	**S9**	0.085	0.565	0.252	0.284	0.289	0.029	1
								**C3**							
**S1**	1	0.762	0.186	<0.0001	0	<0.0001	<0.0001	**S10**	1	0.156	0.007	0.000	0.403	0.014	0.017
**S2**	0.762	1	0.208	0.001	0.004	0	0	**S11**	0.156	1	0.088	0.657	0.48	0.098	0.036
**S3**	0.186	0.208	1	0.797	0.822	0.5	0.183	**S12**	0.007	0.088	1	0.000	0.192	0.537	0.06
**S4**	<0.0001	0.001	0.797	1	0.004	0.000	0.003	**S13**	0.000	0.657	0.000	1	0.558	0.000	0.002
**S5**	0	0.004	0.822	0.004	1	0.000	0.001	**S14**	0.403	0.48	0.192	0.558	1	0.221	0.059
**S6**	<0.0001	0.000	0.5	0.000	0.000	1	0.011	**S8**	0.014	0.098	0.537	0.000	0.221	1	0.047
**S7**	<0.0001	0.000	0.183	0.003	0.001	0.011	1	**S9**	0.017	0.036	0.06	0.002	0.059	0.047	1
								**C4**							
**S1**	1	0.625	0.007	0.177	0.005	0	0	**S10**	1	0.181	0.011	<0.0001	0.531	<0.0001	0.109
**S2**	0.625	1	0.002	0.14	<0.0001	<0.0001	<0.0001	**S11**	0.181	1	0.206	0.043	0.179	0.296	0.427
**S3**	0.007	0.002	1	0.678	0.157	0.068	0.012	**S12**	0.011	0.206	1	0.004	0.03	0.412	0.889
**S4**	0.177	0.14	0.678	1	0.911	0.263	0.113	**S13**	<0.0001	0.043	0.004	1	0.564	<0.0001	0.045
**S5**	0.005	<0.0001	0.157	0.911	1	<0.0001	0.000	**S14**	0.531	0.179	0.03	0.564	1	0.025	0.103
**S6**	0	<0.0001	0.068	0.263	<0.0001	1	0.005	**S8**	<0.0001	0.296	0.412	<0.0001	0.025	1	0.785
**S7**	0	<0.0001	0.012	0.113	0	0.005	1	**S9**	0.109	0.427	0.889	0.045	0.103	0.785	1
								**C5**							
**S1**	1	<0.0001	0.001	0.022	0.014	0.383	0.585	**S10**	1	0.726	0.173	0.093	0.917	0.727	0.022
**S2**	<0.0001	1	<0.0001	0.014	<0.0001	<0.0001	<0.0001	**S11**	0.726	1	0.015	0.009	0.572	0.946	0.001
**S3**	0.001	<0.0001	1	0.586	<0.0001	0.000	<0.0001	**S12**	0.173	0.015	1	0.09	<0.0001	0.001	<0.0001

**S4**	0.022	0.014	0.586	1	0.12	0.027	0.02	**S13**	0.093	0.009	0.09	1	0.001	0.001	0.006
**S5**	0.014	<0.0001	<0.0001	0.12	1	0.004	<0.0001	**S14**	0.917	0.572	<0.0001	0.001	1	0.26	<0.0001
**S6**	0.383	<0.0001	0.000	0.027	0.004	1	0.373	**S8**	0.727	0.946	0.001	0.001	0.26	1	<0.0001
**S7**	0.585	<0.0001	<0.0001	0.02	<0.0001	0.373	1	**S9**	0.022	0.001	<0.0001	0.006	<0.0001	<0.0001	1
**C6**															
**S1**	1	0.002	0.005	0.002	0.519	0.745	<0.0001	**S10**	1	0.096	0.135	0.000	0.003	0.422	0.405
**S2**	0.002	1	0.004	0	0.001	0.011	0.719	**S11**	0.096	1	0.586	<0.0001	0.001	0.006	0.099
**S3**	0.005	0.004	1	<0.0001	0.003	0.342	<0.0001	**S12**	0.135	0.586	1	0.003	0.039	0.169	0.221
**S4**	0.002	0.000	<0.0001	1	0.002	0.041	<0.0001	**S13**	0.000	<0.0001	0.003	1	0.002	<0.0001	<0.0001
**S5**	0.519	0.001	0.003	0.002	1	0.587	<0.0001	**S14**	0.003	0.001	0.039	0.002	1	0.000	0.001
**S6**	0.745	0.011	0.342	0.041	0.587	1	0.006	**S8**	0.422	0.006	0.169	<0.0001	0.000	1	0.783
**S7**	<0.0001	0.719	<0.0001	<0.0001	<0.0001	0.006	1	**S9**	0.405	0.099	0.221	<0.0001	0.001	0.783	1
**C7**															
**S1**	1	<0.0001	0	<0.0001	<0.0001	<0.0001	0	**S10**	1	<0.0001	0.000	0.000	0.000	<0.0001	<0.0001
**S2**	<0.0001	1	<0.0001	0.008	0.002	<0.0001	<0.0001	**S11**	<0.0001	1	<0.0001	<0.0001	<0.0001	<0.0001	0.005
**S3**	0.000	<0.0001	1	<0.0001	<0.0001	<0.0001	<0.0001	**S12**	0.000	<0.0001	1	0.896	0.052	0.001	0.001
**S4**	<0.0001	0.008	<0.0001	1	0	<0.0001	<0.0001	**S13**	0.000	<0.0001	0.896	1	0.083	0.003	0.002
**S5**	<0.0001	0.002	<0.0001	0.000	1	<0.0001	<0.0001	**S14**	0.000	<0.0001	0.052	0.083	1	0.015	0.005
**S6**	<0.0001	<0.0001	<0.0001	<0.0001	<0.0001	1	0.000	**S8**	<0.0001	<0.0001	0.001	0.003	0.015	1	0.037
**S7**	0.000	<0.0001	<0.0001	<0.0001	<0.0001	0.000	1	**S9**	<0.0001	0.005	0.001	0.002	0.005	0.037	1
**C8**															
**S1**	1	0.423	0.65	1	0.339	0.968	0.819	**S10**	1	0.000	0.01	0.138	0.000	<0.0001	0.019
**S2**	0.423	1	0.311	0.056	0.588	0.038	0.124	**S11**	0.000	1	0.000	0.000	0.407	0.025	0.001
**S3**	0.65	0.311	1	0.202	0.088	0.161	0.485	**S12**	0.01	0.000	1	0.001	0.000	<0.0001	0.442
**S4**	1	0.056	0.202	1	0.01	0.873	0.471	**S13**	0.138	0.000	0.001	1	0.000	<0.0001	0.007
**S5**	0.339	0.588	0.088	0.01	1	0.002	0.025	**S14**	0.000	0.407	0.000	0.000	1	0.01	0.001
**S6**	0.968	0.038	0.161	0.873	0.002	1	0.45	**S8**	<0.0001	0.025	<0.0001	<0.0001	0.01	1	0.000
**S7**	0.819	0.124	0.485	0.471	0.025	0.45	1	**S9**	0.019	0.001	0.442	0.007	0.001	0.000	1
**C9**															
**S1**	1	0.069	0.843	0.683	0.863	0.98	0.954	**S10**	1	0.137	0.366	0.967	0.515	0.04	0.196
**S2**	0.069	1	0.004	0.001	0.016	0.11	0.006	**S11**	0.137	1	0.126	0.174	0.89	0.115	0.366
**S3**	0.843	0.004	1	0.034	0.547	0.848	0.719	**S12**	0.366	0.126	1	0.414	0.68	0.04	0.081
**S4**	0.683	0.001	0.034	1	0.738	0.767	0.204	**S13**	0.967	0.174	0.414	1	0.511	0.048	0.248
**S5**	0.863	0.016	0.547	0.738	1	0.907	0.711	**S14**	0.515	0.89	0.68	0.511	1	0.601	0.781
**S6**	0.98	0.11	0.848	0.767	0.907	1	0.937	**S8**	0.04	0.115	0.04	0.048	0.601	1	0.06
**S7**	0.954	0.006	0.719	0.204	0.711	0.937	1	**S9**	0.196	0.366	0.081	0.248	0.781	0.06	1
**C10**															
**S1**	1	0.078	0.73	1	0.985	0.949	0.966	**S10**	1	0.265	0.242	0.676	0.309	0.079	0.781
**S2**	0.078	1	0.008	0.052	0.08	0.01	0.117	**S11**	0.265	1	0.779	0.023	0.606	0.175	0.101
**S3**	0.73	0.008	1	0.682	0.75	0.462	0.741	**S12**	0.242	0.779	1	0.051	0.504	0.502	0.147
**S4**	1	0.052	0.682	1	0.983	0.94	0.964	**S13**	0.676	0.023	0.051	1	<0.0001	<0.0001	0.005

**S5**	0.985	0.08	0.75	0.983	1	0.969	0.953	**S14**	0.309	0.606	0.504	<0.0001	1	<0.0001	0.004
**S6**	0.949	0.01	0.462	0.94	0.969	1	0.917	**S8**	0.079	0.175	0.502	<0.0001	<0.0001	1	0.000
**S7**	0.966	0.117	0.741	0.964	0.953	0.917	1	**S9**	0.781	0.101	0.147	0.005	0.004	0.000	1
								**C11**							
**S1**	1	0.172	0.002	0.029	0.001	0.017	0.718	**S10**	1	<0.0001	0.059	0	0.001	<0.0001	<0.0001
**S2**	0.172	1	0	<0.0001	<0.0001	0.001	0.01	**S11**	<0.0001	1	0.001	<0.0001	0.001	<0.0001	0.001
**S3**	0.002	0	1	<0.0001	0.031	0.001	0	**S12**	0.059	0.001	1	0.01	0.006	0.007	0.002
**S4**	0.029	<0.0001	<0.0001	1	<0.0001	<0.0001	<0.0001	**S13**	0	<0.0001	0.01	1	0	<0.0001	<0.0001
**S5**	0.001	<0.0001	0.031	<0.0001	1	<0.0001	<0.0001	**S14**	0.001	0.001	0.006	0	1	0.281	0.016
**S6**	0.017	0.001	0.001	<0.0001	<0.0001	1	<0.0001	**S8**	<0.0001	<0.0001	0.007	<0.0001	0.281	1	<0.0001
**S7**	0.718	0.01	0.000	<0.0001	<0.0001	<0.0001	1	**S9**	<0.0001	0.001	0.002	<0.0001	0.016	<0.0001	1

C1 - *trans*-resveratrol; C2 - *cis*-resveratrol; C3 - epicatechin; C4 - catechin; C5 - gallic acid; C6 - protocatechuic acid; C7 - caftaric acid; C8 - caffeic acid; C9 - p-coumaric acid; C10 - ferulic acid; C11 - melatonin; S1 - control sample, no bâtonnage, no exogenous yeasts; S2 - *Lachancea thermotolerans* yeasts; S3 - *Saccharomyces cerevisiae* yeasts; S4 - *Torulaspora delbrueckii* yeasts; S5 - *Pichia kluyveri* yeasts; S6 - *Saccharomyces cerevisiae+Kluyveromyces thermotolerans* yeasts; S7 - *Kluyveromyces thermotolerans+Torulaspora delbrueckii+Saccharomyces cerevisiae* yeasts; S8 - control sample, with bâtonnage, no exogenous yeasts; S9 - *Lachancea thermotolerans* yeasts+bâtonnage products; S10 - *Saccharomyces cerevisiae* yeasts+bâtonnage products; S11 - *Torulaspora delbrueckii* yeasts+bâtonnage products; S12 - *Pichia kluyveri* yeasts+bâtonnage products; S13 - *Saccharomyces cerevisiae+Kluyveromyces thermotolerans* yeasts+bâtonnage products; S14 - *Kluyveromyces thermotolerans+Torulaspora delbrueckii+Saccharomyces cerevisiae* yeasts+bâtonnage products. The results are significantly influenced by the applied technology when *p*-value is less than 0.05.

Table S2. Student's t-test for Sauvignon blanc wines.

	**Without bâtonnage products**								**With bâtonnage products**						
	**S15**	**S16**	**S17**	**S18**	**S19**	**S20**	**S21**		**S22**	**S23**	**S24**	**S25**	**S26**	**S27**	**S28**
								**C1**							
**S15**		0.659	1.000	0.818	0.059	0.005	0.040	**S22**		0.635	<0.0001	0.003	0.001	0.001	0.002
**S16**	0.659		0.196	0.689	0.024	0.000	0.010	**S23**	0.635		<0.0001	0.000	0.000	<0.0001	<0.0001
**S17**	1.000	0.196		0.808	0.012	<0.0001	0.004	**S24**	<0.0001	<0.0001		<0.0001	0.001	<0.0001	<0.0001
**S18**	0.818	0.689	0.808		0.250	0.089	0.222	**S25**	0.003	0.000	<0.0001		0.013	0.151	<0.0001
**S19**	0.059	0.024	0.012	0.250		0.032	0.800	**S26**	0.001	0.000	0.001	0.013		0.015	<0.0001
**S20**	0.005	0.000	<0.0001	0.089	0.032		0.025	**S27**	0.001	<0.0001	<0.0001	0.151	0.015		<0.0001
**S21**	0.040	0.010	0.004	0.222	0.800	0.025		**S28**	0.002	<0.0001	<0.0001	<0.0001	<0.0001	<0.0001	
								**C2**							
**S15**		0.696	0.000	0.001	0.435	0.000	0.268	**S22**		<0.0001	0.000	0.357	0.039	0.008	0.015
**S16**	0.696		0.012	0.040	0.551	0.006	0.732	**S23**	<0.0001		0.013	<0.0001	<0.0001	<0.0001	<0.0001
**S17**	0.000	0.012		<0.0001	<0.0001	<0.0001	0.004	**S24**	0	0.013		0.000	0.000	<0.0001	<0.0001
**S18**	0.001	0.040	<0.0001		<0.0001	0.001	0.006	**S25**	0.357	<0.0001	0.000		0.125	0.075	0.153
**S19**	0.435	0.551	<0.0001	<0.0001		<0.0001	0.158	**S26**	0.039	<0.0001	0.000	0.125		0.6	0.343
**S20**	0.000	0.006	<0.0001	0.001	<0.0001		0.001	**S27**	0.008	<0.0001	<0.0001	0.075	0.6		<0.0001
**S21**	0.268	0.732	0.004	0.006	0.158	0.001		**S28**	0.015	<0.0001	<0.0001	0.153	0.343	<0.0001	
								**C3**							
**S15**		0.527	0.573	0.081	0.448	0.053	0.065	**S22**		0.982	0.783	0.203	0.856	0.016	0.236
**S16**	0.527		1.000	0.127	1.000	0.074	0.094	**S23**	0.982		0.771	0.127	0.792	0.008	0.193

**S17**	0.573	1.000		0.196	1.000	0.125	0.168	**S24**	0.783	0.771		0.04	0.421	0.002	0.118
**S18**	0.081	0.127	0.196		0.036	0.573	1.000	**S25**	0.203	0.127	0.04		0.002	0.001	0.704
**S19**	0.448	1.000	1.000	0.036		0.018	0.008	**S26**	0.856	0.792	0.421	0.002		<0.0001	0.151
**S20**	0.053	0.074	0.125	0.573	0.018		0.482	**S27**	0.016	0.008	0.002	0.001	<0.0001		0.106
**S21**	0.065	0.094	0.168	1.000	0.008	0.482		**S28**	0.236	0.193	0.118	0.704	0.151	0.106	
**C4**															
**S15**		0.573	0.448	0.687	0.410	0.410	0.357	**S22**		0.888	0.117	0.346	0.905	0.041	0.267
**S16**	0.573		0.168	0.264	0.201	0.201	0.158	**S23**	0.888		0.012	0.127	0.837	0.022	0.213
**S17**	0.448	0.168		0.158	0.696	0.696	0.613	**S24**	0.117	0.012		0.186	0.442	0.003	0.019
**S18**	0.687	0.264	0.158		0.435	0.435	0.313	**S25**	0.346	0.127	0.186		0.683	0.008	0.054
**S19**	0.410	0.201	0.696	0.435		1.000	1.000	**S26**	0.905	0.837	0.442	0.683		0.115	0.399
**S20**	0.410	0.201	0.696	0.435	1.000		1.000	**S27**	0.041	0.022	0.003	0.008	0.115		0.167
**S21**	0.357	0.158	0.613	0.313	1.000	1.000		**S28**	0.267	0.213	0.019	0.054	0.399	0.167	
**C5**															
**S15**		<0.0001	0.001	<0.0001	<0.0001	0.070	0.527	**S22**		<0.0001	0.201	0.014	<0.0001	0.001	0.028
**S16**	<0.0001		<0.0001	<0.0001	<0.0001	<0.0001	<0.0001	**S23**	<0.0001		<0.0001	<0.0001	<0.0001	<0.0001	<0.0001
**S17**	0.001	<0.0001		<0.0001	<0.0001	0.007	0.808	**S24**	0.201	<0.0001		0.074	0.118	0.088	0.071
**S18**	<0.0001	<0.0001	<0.0001		0.012	<0.0001	0.001	**S25**	0.014	<0.0001	0.074		<0.0001	0.000	0.773
**S19**	<0.0001	<0.0001	<0.0001	0.012		<0.0001	0.001	**S26**	<0.0001	<0.0001	0.118	<0.0001		0.393	0.000
**S20**	0.070	<0.0001	0.007	<0.0001	<0.0001		0.631	**S27**	0.001	<0.0001	0.088	0	0.393		0.000
**S21**	0.527	<0.0001	0.808	0.001	0.001	0.631		**S28**	0.028	<0.0001	0.071	0.773	0.000	0.000	
**C6**															
**S15**		0.052	0.313	0.003	0.527	0.178	0.313	**S22**		0.065	<0.0001	0.001	0.01	0.035	0.02
**S16**	0.052		0.007	0.004	0.042	0.613	0.000	**S23**	0.065		<0.0001	0.000	0.025	0.384	0.069
**S17**	0.313	0.007		0.001	0.158	0.313	<0.0001	**S24**	<0.0001	<0.0001		0.001	0.000	<0.0001	0.000
**S18**	0.003	0.004	0.001		0.003	0.008	0.000	**S25**	0.001	0.000	0.001		0.028	0.001	0.013
**S19**	0.527	0.042	0.158	0.003		0.106	1.000	**S26**	0.01	0.025	0.000	0.028		0.037	0.485
**S20**	0.178	0.613	0.313	0.008	0.106		0.026	**S27**	0.035	0.384	<0.0001	0.001	0.037		0.111
**S21**	0.313	0.000	<0.0001	0.000	1.000	0.026		**S28**	0.02	0.069	0.000	0.013	0.485	0.111	
**C7**															
**S15**		<0.0001	0.001	0.000	<0.0001	0.632	0.264	**S22**		0.002	0.09	0.298	0.977	0.001	0.803
**S16**	<0.0001		0.268	0.036	0.000	0.010	0.028	**S23**	0.002		0.73	0.003	0.104	<0.0001	0.181
**S17**	0.001	0.268		0.340	0.003	0.009	0.023	**S24**	0.09	0.73		0.136	0.229	0.01	0.328
**S18**	0.000	0.036	0.340		0.004	0.005	0.016	**S25**	0.298	0.003	0.136		0.8	0	1
**S19**	<0.0001	0.000	0.003	0.004		0.001	0.005	**S26**	0.977	0.104	0.229	0.8		0.131	0.864
**S20**	0.632	0.010	0.009	0.005	0.001		0.405	**S27**	0.001	<0.0001	0.01	0.000	0.131		0.128
**S21**	0.264	0.028	0.023	0.016	0.005	0.405		**S28**	0.803	0.181	0.328	1	0.864	0.128	
**C8**															
**S15**		0.275	0.018	0.047	0.074	0.265	0.542	**S22**		0.003	0.107	0.368	0.041	0.505	0.058
**S16**	0.275		0.208	0.530	0.574	0.595	0.380	**S23**	0.003		<0.0001	0.000	<0.0001	<0.0001	<0.0001
**S17**	0.018	0.208		0.004	0.204	<0.0001	0.002	**S24**	0.107	<0.0001		0.003	0.000	0.007	0.007

**S18**	0.047	0.530	0.004		1.000	0.001	0.008	**S25**	0.368	0	0.003		0.031	0.028	0.002
**S19**	0.074	0.574	0.204	1.000		0.077	0.051	**S26**	0.041	<0.0001	0.000	0.031		0.001	0.000
**S20**	0.265	0.595	<0.0001	0.001	0.077		0.246	**S27**	0.505	<0.0001	0.007	0.028	0.001		0.002
**S21**	0.542	0.380	0.002	0.008	0.051	0.246		**S28**	0.058	<0.0001	0.007	0.002	0.000	0.002	
**C9**															
**S15**	1	1.000	0.051	0.503	0.759	0.037	0.122	**S22**		0.015	0.000	0.276	0.819	0.000	0.001
**S16**	1.000	1	0.022	0.436	0.666	0.010	0.082	**S23**	0.015		0.003	0.015	0.035	0.000	0.046
**S17**	0.051	0.022	1	0.141	0.012	1.000	0.775	**S24**	0	0.003		<0.0001	0.001	<0.0001	0.018
**S18**	0.503	0.436	0.141	1	0.547	0.108	0.288	**S25**	0.276	0.015	<0.0001		0.391	<0.0001	0.001
**S19**	0.759	0.666	0.012	0.547	1	0.002	0.083	**S26**	0.819	0.035	0.001	0.391		0.004	0.005
**S20**	0.037	0.010	1.000	0.108	0.002	1	0.749	**S27**	0.000	0.000	<0.0001	<0.0001	0.004		<0.0001
**S21**	0.122	0.082	0.775	0.288	0.083	0.749	1	**S28**	0.001	0.046	0.018	0.001	0.005	<0.0001	
**C10**															
**S15**		0.340	0.305	0.092	0.918	0.058	0.138	**S22**		0.005	0.004	0.173	0.568	0.158	0.004
**S16**	0.340		0.444	0.248	0.607	0.105	0.201	**S23**	0.005		0.34	0.6	0.002	0.003	0.353
**S17**	0.305	0.444		0.696	0.357	0.828	0.650	**S24**	0.004	0.34		0.779	0.001	<0.0001	1
**S18**	0.092	0.248	0.696		0.335	0.283	0.336	**S25**	0.173	0.6	0.779		0.124	0.308	0.779
**S19**	0.918	0.607	0.357	0.335		0.159	0.186	**S26**	0.568	0.002	0.001	0.124		0.034	0.001
**S20**	0.058	0.105	0.828	0.283	0.159		0.718	**S27**	0.158	0.003	<0.0001	0.308	0.034		<0.0001
**S21**	0.138	0.201	0.650	0.336	0.186	0.718		**S28**	0.004	0.353	1	0.779	0.001	<0.0001	
**C11**															
**S15**	1	<0.0001	<0.0001	<0.0001	<0.0001	<0.0001	<0.0001	**S22**		1.000	0.542	0.861	0.661	0.468	0.025
**S16**	<0.0001	1	0.002	0.364	0.025	<0.0001	0.235	**S23**	1.000		0.048	0.345	0.238	0.174	0.000
**S17**	<0.0001	0.002	1	0.008	0.473	<0.0001	0.001	**S24**	0.542	0.048		0.101	0.667	0.709	0.001
**S18**	<0.0001	0.364	0.008	1	0.025	<0.0001	0.896	**S25**	0.861	0.345	0.101		0.430	0.258	0.000
**S19**	<0.0001	0.025	0.473	0.025	1	<0.0001	0.010	**S26**	0.661	0.238	0.667	0.430		0.551	0.002
**S20**	<0.0001	<0.0001	<0.0001	<0.0001	<0.0001	1	<0.0001	**S27**	0.468	0.174	0.709	0.258	0.551		0.011
**S21**	<0.0001	0.235	0.001	0.896	0.010	<0.0001	1	**S28**	0.025	0.000	0.001	0.000	0.002	0.011	

C1 - *trans*-resveratrol; C2 - *cis*-resveratrol; C3 - epicatechin; C4 - catechin; C5 - gallic acid; C6 - protocatechuic acid; C7 - caftaric acid; C8 - caffeic acid; C9 - *p*-coumaric acid; C10 - ferulic acid; C11 - melatonin; S15 - control sample, no bâtonnage, no exogenous yeasts; S16 - *Lachancea thermotolerans* yeasts; S17 - *Saccharomyces cerevisiae* yeasts; S18 - *Torulaspora delbrueckii* yeasts; S19 - *Pichia kluyveri* yeasts; S20 - *Saccharomyces cerevisiae+Kluyveromyces thermotolerans* yeasts; S21 - *Kluyveromyces thermotolerans+Torulaspora delbrueckii+Saccharomyces cerevisiae* yeasts; S22 - control sample, with bâtonnage, no exogenous yeasts; S23 - *Lachancea thermotolerans* yeasts+bâtonnage products; S24 - *Saccharomyces cerevisiae* yeasts+bâtonnage products; S25 - *Torulaspora delbrueckii* yeasts+bâtonnage products; S26 - *Pichia kluyveri* yeasts+bâtonnage products; S27 - *Saccharomyces cerevisiae+Kluyveromyces thermotolerans* yeasts+bâtonnage products; S28 - *Kluyveromyces thermotolerans+Torulaspora delbrueckii+Saccharomyces cerevisiae* yeasts+bâtonnage products. The results are significantly influenced by the applied technology when *p*-value is less than 0.05.

Table S3. T-test between samples without and with bâtonnage.

Pairs	C1	C2	C3	C4	C5	C6	C7	C8	C9	C10	C11
**Aligoté+Fetească albă**											

**S1-S8**	0.006	0.000	<0.0001	0.001	0.140	<0.0001	0.000	<0.0001	0.004	0.003	0.002
**S2-S9**	0.014	0.045	0.008	0.018	<0.0001	0.127	<0.0001	<0.0001	<0.0001	0.000	0.000
**S3-S10**	0.003	0.006	0.746	0.072	0.055	0.158	<0.0001	<0.0001	<0.0001	0.002	0.004
**S4-S11**	0.747	0.021	0.131	0.486	0.031	<0.0001	<0.0001	<0.0001	<0.0001	0.002	<0.0001
**S5-S12**	0.405	0.072	0.018	0.448	<0.0001	0.007	<0.0001	<0.0001	0.000	0.007	0.031
**S6-S13**	0.000	1.000	0.158	<0.0001	0.000	0.000	<0.0001	<0.0001	0.003	<0.0001	<0.0001
**S7-S14**	0.542	0.026	0.051	0.268	<0.0001	0.001	<0.0001	<0.0001	0.008	0.003	0.000
					**Sauvignon blanc**						
**S15-S22**	0.001	<0.0001	0.340	1.000	<0.0001	0.000	<0.0001	<0.0001	<0.0001	0.001	<0.0001
**S16-S23**	<0.0001	<0.0001	0.106	0.448	<0.0001	<0.0001	<0.0001	<0.0001	<0.0001	<0.0001	0.001
**S17-S24**	0.001	<0.0001	0.106	0.001	0.087	0.001	<0.0001	<0.0001	<0.0001	0.001	<0.0001
**S18-S25**	0.082	<0.0001	0.018	0.007	<0.0001	0.045	<0.0001	<0.0001	<0.0001	0.082	0.040
**S19-S26**	0.001	0.000	0.001	0.288	<0.0001	0.003	<0.0001	<0.0001	<0.0001	0.001	0.002
**S20-S27**	<0.0001	<0.0001	0.482	0.718	0.840	0.000	<0.0001	<0.0001	<0.0001	<0.0001	<0.0001
**S21-S28**	<0.0001	0.000	0.246	0.656	0.012	0.000	<0.0001	<0.0001	<0.0001	<0.0001	0.039

C1 - *trans*-resveratrol; C2 - *cis*-resveratrol; C3 - epicatechin; C4 - catechin; C5 - gallic acid; C6 - protocatechuic acid; C7 - caftaric acid; C8 - caffeic acid; C9 - p-coumaric acid; C10 - ferulic acid; C11 - melatonin. S1-S14 - Aligoté+Fetească albă: S1 - control sample, no bâtonnage, no exogenous yeasts; S2 - *Lachancea thermotolerans* yeasts; S3 - *Saccharomyces cerevisiae* yeasts; S4 - *Torulaspora delbrueckii* yeasts; S5 - *Pichia kluyveri* yeasts; S6 - *Saccharomyces cerevisiae+Kluyveromyces thermotolerans* yeasts; S7 - *Kluyveromyces thermotolerans+Torulaspora delbrueckii+Saccharomyces cerevisiae* yeasts; S8 - control sample, with bâtonnage, no exogenous yeasts; S9 - *Lachancea thermotolerans* yeasts+bâtonnage products; S10 - *Saccharomyces cerevisiae* yeasts+bâtonnage products; S11 - *Torulaspora delbrueckii* yeasts+bâtonnage products; S12 - *Pichia kluyveri* yeasts+bâtonnage products; S13 - *Saccharomyces cerevisiae+Kluyveromyces thermotolerans* yeasts+bâtonnage products; S14 - *Kluyveromyces thermotolerans+Torulaspora delbrueckii+Saccharomyces cerevisiae* yeasts+bâtonnage products.

S15-S28 - Sauvignon blanc: S15 - control sample, no bâtonnage, no exogenous yeasts; S16 - *Lachancea thermotolerans* yeasts; S17 - *Saccharomyces cerevisiae* yeasts; S18 - *Torulaspora delbrueckii* yeasts; S19 - *Pichia kluyveri* yeasts; S20 - *Saccharomyces cerevisiae+Kluyveromyces thermotolerans* yeasts; S21 - *Kluyveromyces thermotolerans+Torulaspora delbrueckii+Saccharomyces cerevisiae* yeasts; S22 - control sample, with bâtonnage, no exogenous yeasts; S23 - *Lachancea thermotolerans* yeasts+bâtonnage products; S24 - *Saccharomyces cerevisiae* yeasts+bâtonnage products; S25 - *Torulaspora delbrueckii* yeasts+bâtonnage products; S26 - *Pichia kluyveri* yeasts+bâtonnage products; S27 - *Saccharomyces cerevisiae+Kluyveromyces thermotolerans* yeasts+bâtonnage products; S28 - *Kluyveromyces thermotolerans+Torulaspora delbrueckii+Saccharomyces cerevisiae* yeasts+bâtonnage products. The results are significantly influenced by the applied technology when p-value is less than 0.05.
